# Classification of heterogeneous microarray data by maximum entropy kernel

**DOI:** 10.1186/1471-2105-8-267

**Published:** 2007-07-26

**Authors:** Wataru Fujibuchi, Tsuyoshi Kato

**Affiliations:** 1 National Institute of Advanced Industrial Science and Technology (AIST), Computational Biology Research Center, 2-42 Aomi, Koto-ku, Tokyo 135-0064, Japan; 2 Graduate School of Frontier Sciences, University of Tokyo, 5-1-5 Kashiwanoha, Kashiwa, Chiba 277-8562, Japan

## Abstract

**Background:**

There is a large amount of microarray data accumulating in public databases, providing various data waiting to be analyzed jointly. Powerful kernel-based methods are commonly used in microarray analyses with support vector machines (SVMs) to approach a wide range of classification problems. However, the standard vectorial data kernel family (linear, RBF, etc.) that takes vectorial data as input, often fails in prediction if the data come from different platforms or laboratories, due to the low gene overlaps or consistencies between the different datasets.

**Results:**

We introduce a new type of kernel called maximum entropy (ME) kernel, which has no pre-defined function but is generated by kernel entropy maximization with sample distance matrices as constraints, into the field of SVM classification of microarray data. We assessed the performance of the ME kernel with three different data: heterogeneous kidney carcinoma, noise-introduced leukemia, and heterogeneous oral cavity carcinoma metastasis data. The results clearly show that the ME kernel is very robust for heterogeneous data containing missing values and high-noise, and gives higher prediction accuracies than the standard kernels, namely, linear, polynomial and RBF.

**Conclusion:**

The results demonstrate its utility in effectively analyzing promiscuous microarray data of rare specimens, e.g., minor diseases or species, that present difficulty in compiling homogeneous data in a single laboratory.

## Background

Microarray has become a standard tool in many biological studies. Typically, classification analyses, where gene expressions of distinct biological groups are compared and classified according to their gene expression characteristics, are frequently performed in various clinical situations such as tumor diagnosis [1,2], anti-cancer drug response analysis [3,4], and prognosis analysis [5,6]. Kernel methods [7] play important roles in such disease analyses, especially when classifying data with support vector machines (SVMs) [8] based on the feature or marker genes that are correlated with the characteristics of the groups. In most of those studies, only standard kernels such as linear, polynomial, and RBF (radial basis function), which take vectorial data as input, have been popularly used and generally successful.

Other than the above *vectorial data kernel *family, there is another family called *structured data kernel *family that has been studied in many other fields including bioinformatics and machine learning [9-12]. The structured data kernel family conveys structural or topological information with or without numerical data as input to describe data. For example, the string kernel for text classification [9], the marginalized count kernel [10] for biological sequences, the diffusion kernel [11] and the maximum entropy (ME) kernel [12] for graph structures are well known in the biological field.

In microarray analysis, one of the main issues that hamper accurate and realistic predictions is the lack of repeat experiments, often due to financial problems or rarity of specimens such as minor diseases. Utilization of public or old data together with one's current data could solve this problem; many studies combining several microarray datasets have been performed [13-15]. However, due to the low gene overlaps and consistencies between different datasets, the vectorial data kernels are often unsuccessful in classifying data from various datasets if naïvely integrated [14].

**Figure 1:**
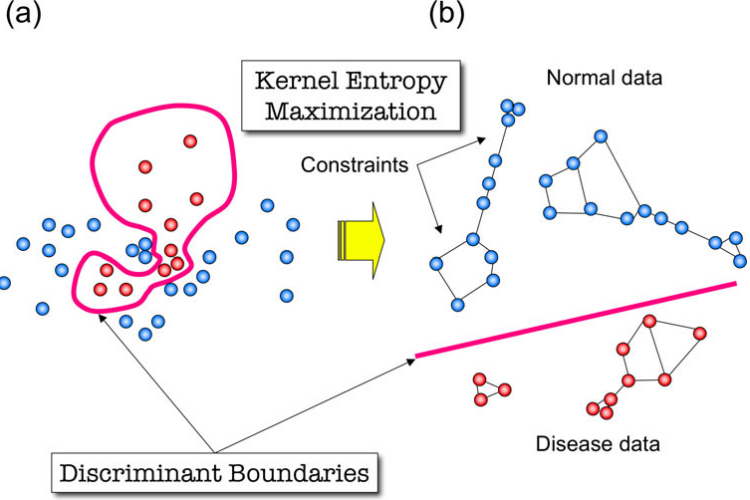
**Maximum entropy kernel for heterogeneous data**. Samples and their distance constraints in the feature space are drawn schematically as graph nodes and edges, respectively. (a) The heterogeneous data are entangled in the feature space, making it difficult to find the discriminant boundary. (b) After kernel entropy maximization, the distances among samples are expanded in the feature space under constraints that hold only similar samples closely, making it easier to find the discriminant boundary.

Among the structured data kernels, the ME kernel can take any distance data as input, and is thus applicable to vectorial data as well when converted into the Euclidean or other types of distance relationships among vectors. Since the ME kernel increases the distances among different sample vectors (or samples hereafter), while keeping similar samples in close distance, discriminative boundaries may be found more explicitly than in the case of the vectorial kernels due to the sparse distribution of heterogeneous samples (Figure 1). Furthermore, the ME kernel has, unlike the RBF kernel, a special property of excluding arbitrary gene values composing vectorial data in calculating the distances among samples. Hence, by ignoring only spurious gene values in each sample without deleting those genes entirely from a dataset, the ME kernel can effectively utilize gene expression information in heterogeneous data containing mosaic-like missing or noisy values.

This paper is constructed as follows. We first show how the ME kernel can effectively work in heterogeneous microarray data using the Euclidean distance among sample vectors. Then, we show the unique and powerful noise reduction ability of the ME kernel in microarray data. Finally, we demonstrate that the ME kernel performs better than the standard kernels in classifying practical microarray data, namely, squamous cell carcinoma metastasis in the oral cavity.

## Results

**Figure 2:**
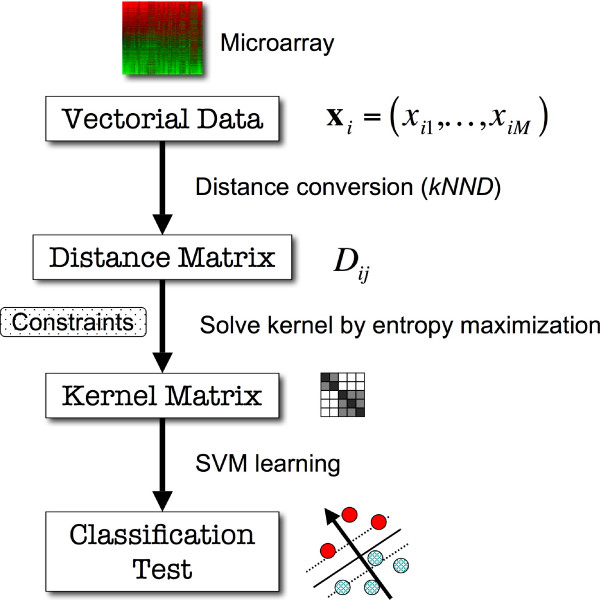
**Schematic view of the entire process of microarray classification in the ME kernel algorithm**. The input vectorial data are first converted into distance matrix to provide constraints *D*_*ij*_. Then, entropy of a kernel matrix is maximized under the constraints, generating an optimal kernel matrix that is guaranteed to be positive semidefinite. Then, the SVM learns the classification boundary from the kernel matrix and classifies test samples.

We describe herein the classification performance of the ME kernel, compared to that of the three standard kernels, linear, polynomial and RBF. We also test two types of distance-based kernels, EKM and Saigo [16,17], for comparison. The schematic view of the entire analysis process is shown in Figure 2. Note that the RBF kernel also uses Euclidean distance as the metric of sample (dis-) similarities but cannot use the *k*-nearest neighbor gene distance (*k*NND) since it violates the positive semidefiniteness of kernels.

We first use *heterogeneous *kidney carcinoma data to confirm the ME kernel's superior discrimination ability against a highly mixed heterogeneous dataset. Then, we demonstrate the ME kernel's interesting denoising ability based on *k*NND using *homogeneous *leukemia microarray data with artificial noise. Finally, we further apply the ME kernel with *k*NND denoising to a more practical problem, i.e., *heterogeneous *data of squamous cell carcinoma metastasis in the oral cavity, to assess its total performance.

### Data normalization and classification analysis

Before testing the performance, all the data are properly normalized by being first log-transformed, and then scaled to mean 0 and standard deviation 1 (i.e., Z-normalization) in each sample and then each gene. All the normalized datasets are available for free at our Internet server [18]. Also the ME program that runs on Linux OS is available upon request. Many genes have a large number of missing values because heterogeneous data are combined; thus, we adopt a simple imputation method that all the missing values are replaced with the mean value, i.e., 0. Input genes that show high correlation to class labels, or feature genes, are selected by the standard two sample t-statistics [19] in each iteration of the leave-one-out cross-validation (LOOCV) test. The distance constraint matrices (*D*_*ij*_) are also generated from the same feature genes. If a sample contains missing values, we again adopt a simple imputation; we replace the one-dimensional Euclidean distance (*x*_*ih*_*- x*_*jh*_)^2 ^with 2 if *x*_*ih *_or *x*_*jh *_is missing. The six kernels are tested with SVMs to analyze their classification performance with various numbers of feature genes and various parameters described in Methods. The maximum accuracy among the tested parameters for each number of feature genes is recorded as the accuracy for each kernel.

### Heterogeneous kidney carcinoma data

The human kidney data of normal tissues and renal clear carcinoma tissues are collected from the public gene expression database, GEO-Gene Expression Omnibus [20]. This dataset is comprised of ten platforms, two of which are spotted DNA/cDNA arrays and eight are variations of Affymetrix-type oligonucleotide arrays. To uniformly analyze the array data from different platforms, we converted as many probe names as possible to UniGene identifiers and combined all the data. The total number of UniGenes in the integrated table is as large as 54,674, all of which contain missing values in some platforms; i.e., there are no genes common to all platforms. The total number of normal and carcinoma data is 100 (62 normal and 38 carcinoma). The characteristics of each data in the composite dataset, such as platform ID, array type, number of data, and experimental comments, are shown in Table 1.

**Table 1:** Organization of heterogeneous kidney carcinoma dataset

**Platform**	**Array type**	**#Normal/Carcinoma**	**Brief comments**
GPL9	Spotted, DNA/cDNA	10/10	Renal clear cell carcinoma, primary tumor [30]
GPL10	Spotted, DNA/cDNA	10/10	Renal clear cell carcinoma, primary tumor [30]
GPL91	Affymetrix, oligo	14/0	Large-scale analysis of the human tran-scriptome (HG-U95A) kidney [31]; Normal human tissue expression profiling (HG-U95A) kidney [32]; Kidney transplant rejection expression profiling kidney normal donor [33]
GPL96	Affymetrix, oligo	10/9	Large-scale analysis of the human tran-scriptome (HG-U133A) kidney [34]; Renal clear cell carcinoma (HG-U133A) [35]
GPL97	Affymetrix, oligo	8/9	Renal clear cell carcinoma (HG-U133B) [35]
GPL92	Affymetrix, oligo	2/0	Normal human tissue expression profiling (HG-U95B) kidney [32]
GPL93	Affymetrix, oligo	2/0	Normal human tissue expression profiling (HG-U95C) kidney [32]
GPL94	Affymetrix, oligo	2/0	Normal human tissue expression profiling (HG-U95D) kidney [32]
GPL95	Affymetrix, oligo	2/0	Normal human tissue expression profiling (HG-U95E) kidney [32]
GPL 1074	Affymetrix, oligo	2/0	Large-scale analysis of the human tran-scriptome (GNFlb) kidney [34]

Platform IDs, array types, number of data, and experimental comments are shown.

**Figure 3:**
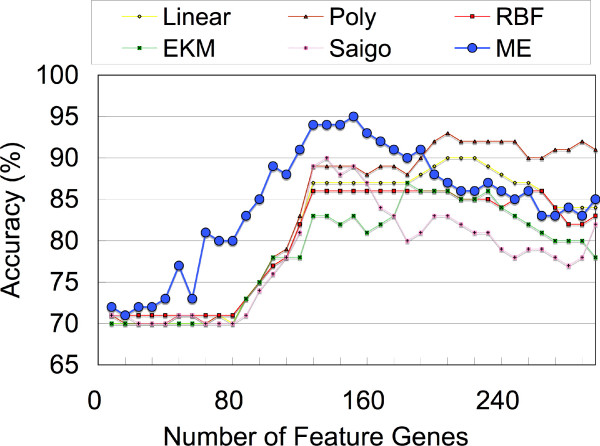
**Classifications of heterogeneous renal carcinoma data with standard and ME kernels**. In most cases, the ME kernel shows much better performance than the linear, polynomial, and RBF kernels and the two distance-based kernels for various numbers of feature genes.

Classification analysis is performed between normal and carcinoma data. The results of the LOOCV test of 100 samples against various numbers (8–296; increasing 8 genes at each step due to computational limitations) of feature genes are plotted in Figure 3. The figure shows typical prediction curves, namely, accuracy increases with increasing number of feature genes, plateaus at some region, and decreases. Clearly, the ME kernel performs much better in all cases than the other five kernels for small numbers of feature genes (8–192). As regards accuracy, the ME kernel records maximum accuracies as high as 95.0 (89.5/98.4 sensitivity/specificity)% for 152 of feature genes. Statistically, the accuracies of the ME kernel are superior to those of the other five kernels in 64.9% of the tested points (8–296) of feature genes. This percentage increases to 95.8% when accuracies are limited only to the increase and plateau regions (8–192) of the ME kernel.

### kNND denoising for AML and ALL data

Acute myeloid leukemia (AML) and acute lymphoblastic leukemia (ALL) data for cancer subtype classification have been reported by Golub *et al*. [1]. There are 72 samples (47 AML and 25 ALL), all of which are quite homogeneous and of good quality, and are thus suitable for artificial noise experiments. To assess the denoising ability of our ME kernel, we first replace the *ν*_add _× 100% of original data in a gene expression profile with artificial white noise, i.e., the noise is added according to a normal distribution model with a mean of 0 and a standard deviation of twice that of each gene value distribution in the original dataset. Then, we extract 50 feature genes from the training dataset for each iteration of the LOOCV test by standard t-statistics.

As the control experiments using linear and RBF kernels, the standard singular value decomposition (SVD) denoising method is applied to reduce noise immediately after the noise is introduced. In the SVD denoising, three levels of noise removals by dfferent cumulative proportions, 85, 90, and 95%, of eigenvalues are explored. For the ME kernel, the *k*NND denoising method with the following noise level settings is applied. First, raw noise that is assumed to internally exist in the original data is arbitrarily set at *ν*_raw _= 0.05. Then, we define the total noise level as the sum of the raw noise and the above artificially added noise, *ν*_add_. For example, if 10% noise is added, the total noise level is *ν*_raw _+ *ν*_add _= 0.05 + 0.1 = 0.15, and (1 - 0.15)^2 ^× 100 = 72.3% of the nearest distance genes out of the feature gene set are considered in calculating the *k*NNDs between samples (see Methods).

**Figure 4:**
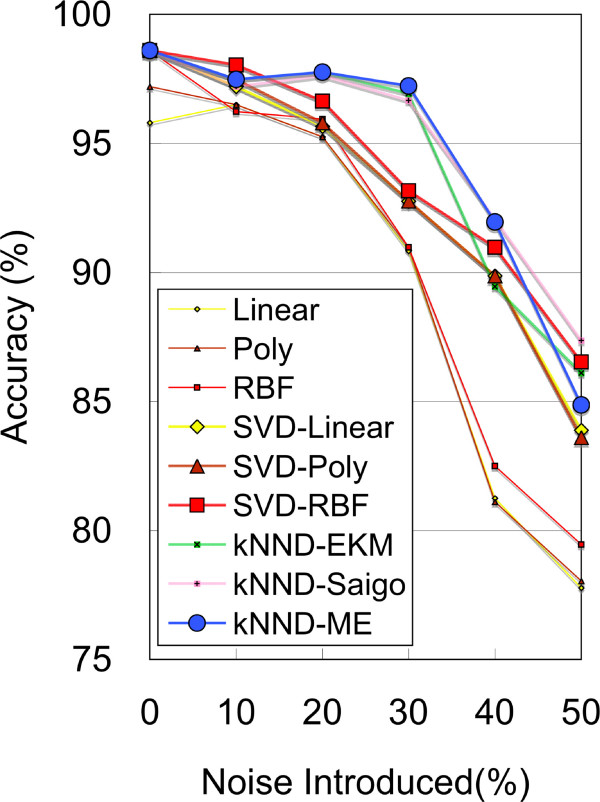
**AML/ALL classification with artificial noise**. The accuracies of standard linear and RBF kernels decrease with increasing noise levels, even with SVD denoising applied, while those of ME and other distance-based kernels with *k*NND denoising are sustained at high levels at 10–40% noise levels

We repeat the above random noise-adding test ten times and average the highest accuracies among various parameter combinations. The results are shown in Figure 4. The artificial noise added is within the range of 0–50%. Since the raw data are quite homogeneous, all kernels except linear and polynomial show the same prediction accuracy of 98.6% when no noise is added. This value decreases gradually with increasing noise levels (10–50%) for the vectorial kernels; for example, the accuracies of the RBF kernel decrease in the order of 96.2, 95.9, 91.0, 82.5, and 79.5%. SVD denoising boosts up these accuracies to 98.0, 96.6, 93.2, 91.0, and 86.5%, respectively. The linear and polynomial kernels also show similar accuracies to the RBF kernel when SVD denoising is used.

Interestingly and surprisingly, the three *k*NND-distance-based methods show high accuracies; for example, the *k*NND-ME kernel has an accuracy of 97.8% even at 20% noise level and maintains high accuracies of 97.2 and 92.0% at 30–40% noise levels. The EKM and Saigo kernels using *k*NND-distance also show similar accuracies to the *k*NND-ME kernel. To verify our results, we extensively analyzed the same data with various parameters including many cumulative proportions in the SVD but obtained similar tendencies, confirming the superior denoising ability of the *k*NND-based method [21].

### Heterogeneous oral cavity carcinoma metastasis data

We further analyze the total performance of the *k*NND-ME method with a more practical problem-heterogeneous oral cavity carcinoma metastasis data. The data consist of two GEO datasets (GSE2280 and GSE3524) from different authors [22,23]. One dataset (GSE2280) is derived from primary squamous cell carcinoma dataset of the oral cavity [22], containing 14 metastasis (samples from lymph node tissues are excluded) and eight non-metastasis samples. The other oral squamous cell carcinoma dataset (GSE3524) is comprised of nine metastasis and nine non-metastasis samples (two of stage-unknown samples are excluded) [23]. Both are from the same platform, Affymetrix HG-U133A, where 22,283 genes are analyzed. The size of each dataset is too small and not suitable for SVM classification if analyzed separately. However, combining the two datasets, we obtain as many as 23 metastasis and 17 non-metastasis samples, making it possible to carry out the classification analysis.

**Figure 5:**
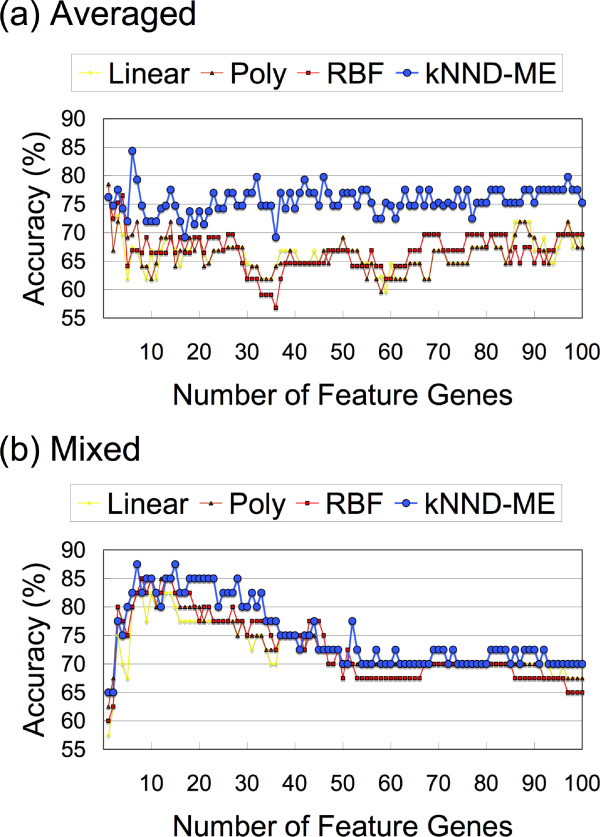
**Oral cavity carcinoma metastasis classification**. Prediction of metastasis by SVMs is performed with gene expression data of squamous cell carcinoma of the oral cavity. Classification accuracies of three kernels, i.e., linear, polynomial, RBF, and ME with *k*NND denoising, are compared. Accuracies are measured by (a) predicting each dataset separately and averaged, and (b) predicting the mixed dataset.

The results of the LOOCV test of the 40 samples against various numbers (1–100; increasing one gene at each step) of feature genes with four different kernels, namely, linear, polynomial, RBF, and ME with *k*NND denoising (*k*NND-ME), are shown in Figure 5. In the *k*NND-ME kernel, five different noise levels, *ν *= 0 (no noise), 0.05, 0.1, 0.15, and 0.2 are evaluated. For comparison, we also classify the two datasets separately and average the accuracies (Figure 5a). The results clearly show that the *k*NND-ME kernel surpasses the other kernels in both averaged and mixed datasets. Statistically, the accuracies of the *k*NND-ME kernel are superior to those of the other three kernels in the averaged and the mixed datasets in 98% and 48%, respectively, of all the tested points. The difference in accuracy is much greater in the averaged dataset than in the mixed dataset. The mean differences between the *k*NND-ME kernel and either of the linear, polynomial or RBF kernel with highest accuracy at each point in the averaged and the mixed datasets are 7.7% and 1.4%, respectively. The accuracies increase and plateau at around 3–30 feature genes in the mixed dataset, while no clear increase or plateau is found for the averaged dataset. The overall maximum accuracy of 87.5 (91.3/82.4 or 82.6/94.1 sensitivity/specificity)% is observed for the *k*NND-ME kernel at two points, 7 and 15 feature genes, in the mixed dataset. Those accuracies are obtained with *ν *= 0 and 0.05 denoising parameters. The result also indicates that the *k*NND-ME kernel shows more stable and higher accuracies than the other kernels for large numbers of feature genes.

Incidentally, the top 15 feature genes that show the highest average ranks by t-statistics in the LOOCV test and that are considered to be associated with oral carcinoma metastasis are: HFE (AF150664), FLJ12529 (NM_024811), CXorf56 (NM_022101), HEATR1 (NM_018072), MGAM (NM_004668), APOL3 (NM_014349), PYY2 (NM_021093), RBP3 (J03912), UBE2V1 (NM_003349, NM_021988, NM_022442, NM_199144, NM_199203), KCNJ15 (U73191), GLS (AB020645), ARHGEF3 (NM_019555), MDM1 (NM_020128), ZC3H13 (AL136745), and C9orf16 (NM_024112).

We further investigate the effect of the SVD denoising when applied to the mixed dataset before learning and classification. Table 2 summarizes the results of using all the six types of kernels for raw and SVD pre-denoised data. The accuracies are averaged in each of the ten gene windows. In the SVD denoising, three levels of noise removals (85, 90, and 95% of cumulative proportions), which are the same as the AML-ALL experiment, are tested.

**Table 2:** Range accuracies for the mixed oral cavity carcinoma metastasis dataset

		**Range of Number of Feature Genes**									
**Kernel**	**Noise**	**1–10 **	**11 – 20 **	**21 – 30 **	**31 – 40 **	**41 – 50 **	**51 – 60 **	**61 – 70 **	**71–80 **	**81 – 90**	**91 – 100 **
Linear	raw	73.8	79.3	77.0	73.5	72.8	70.0	70.0	70.0	70.0	68.5
	SVD	66.3	76.8	74.0	74.8	75.0	76.3	77.5	77.5	77.5	77.5
Polynomial	raw	77.8	81.8	77.3	74.3	72.8	70.0	70.0	70.0	70.0	67.8
	SVD	68.0	79.5	79.0	76.3	75.0	76.0	77.5	77.5	77.5	77.5
RBF	raw	77.0	82.3	78.0	75.8	73.5	68.3	68.5	70.0	68.8	66.5
	SVD	68.0	78.5	79.0	**81.8**	**80.8**	**80.3**	79.5	77.8	78.8	79.0
EKM	raw	74.5	78.5	76.8	72.3	67.3	66.8	65.5	65.5	65.0	65.0
	SVD	68.8	80.0	79.0	**81.8**	**80.8**	**80.3**	80.0	79.5	**81.0**	80.3
Saigo	raw	77.5	81.5	80.3	73.5	70.3	70.0	69.0	68.3	68.0	67.5
	SVD	68.0	77.8	77.5	76.0	75.0	76.8	77.8	78.3	78.0	78.8
*k*NND-ME	raw	**78.5**	**84.0**	**82.8**	77.8	73.3	71.3	70.8	70.5	72.0	70.3
	SVD	69.5	78.5	79.0	79.5	77.5	79.0	**80.5**	**80.0**	80.3	**83.3**

Maximum accuracy in each range of feature gene number is shown in bold.

Although a sufficient number of genes (a total of 22,283 genes) are used for SVD denoising, the denoised dataset does not significantly improve the raw accuracies in small numbers (≤ 30) of feature genes, where the overall maximum range accuracy (84.0%) exists. SVD denoising affects only large numbers (≥ 31) of feature genes. This is probably related to the property of SVD denoising that affects the ratio of information to noise content. Further analysis is needed to understand the full property of the SVD denoising method. In summary, the maximum accuracy (87.5%) of the *k*NND-ME kernel in raw data is not improved by SVD denoising (data not shown).

## Discussion

Using kidney carcinoma data, we show that the ME kernel generally gives better classification results for heterogeneous microarray datasets than the three vectorial data kernels, linear, polynomial and RBF. As an alternative approach using vectorial data kernels, it is theoretically possible to train multiple SVMs for all distinct sub-data contained in the composite dataset. However, this approach has practical diffculties in that (i) there are too many heterogeneous sub-data, (ii) some sub-data contain only a few samples, and (iii) some sub-data contain all positive (or negative) samples. The SVMs cannot be trained properly with only a few samples or data with one-sided (positive or negative) labels. In addition, if we do not know the origin (i.e., platform) of the test samples, it would be diffcult to determine which SVMs should be used for the classification. The ME kernel is much simpler yet quite flexible in this regard.

Another remarkable property of our ME kernel is that the generated kernel matrices always hold positive semidefiniteness, even when the distance matrices for input to our optimization algorithm violate the triangle inequalities. This allows one to arbitrarily choose genes from among a set of feature genes to build the distance matrices in a distance-by-distance fashion. Utilizing this property, we devised the *k*NND denoising method for the distance-based kernels, which show better performance than the linear, polynomial and RBF kernels for leukemia data, even though the data are pre-denoised by SVD. This is quite important in a situation where there are few or heterogeneous samples where SVD may not work properly for denoising because the quality of the eigenvalue decomposition depends on the number of homogeneous samples. Since the *k*NND denoising method only concerns the set of genes between sample pairs, it seems quite robust with regard to the number of samples or the degree of heterogeneity.

Furthermore, the results of kidney carcinoma and oral cavity carcinoma metastasis data in Figure 3 and Table 2 clearly show that the accuracies of the ME kernel exceed those of the other two distance-based kernels, EKM and Saigo. However, in the AML-ALL data shown in Figure 4, the ME kernel and the other two distance-based methods show similar accuracies although all of them use the same *k*NND distance data. From these observations, we can conclude that the entropy maximization process works favorably for 'heterogeneous' data and allows SVMs to find the discriminant boundaries more easily than the other two distance-based methods, EKM and Saigo.

It is also important to point out that combining similar but distinct data in the microarray analysis may enhance the diagnosis of cancer or other diseases. As shown in our example of metastasis prediction for oral squamous cell carcinoma, each dataset contains only around 20 samples, which is not suitable for training of good SVM predictors, especially in the case of the vectorial data kernel family (see Figure 5a). When the datasets are combined, however, our *k*NND-ME kernel demonstrates higher and more robust classification performance than the linear, polynomial, and RBF kernels and even the other two distance-based kernels, regardless of SVD denoising.

## Conclusion

We conclude that the ME kernel-based SVM classification method will generally be useful for the analysis of promiscuous microarray data of rare specimens, e.g., minor diseases or species, that present difficulty in compiling homogeneous data in a single laboratory.

## Methods

In this section, we begin with a preliminary explanation of kernel methods. Then, we describe the ME kernel in terms of its basic and advantageous properties for use with heterogeneous data.

### Properties of kernel methods

Kernels are numerical expressions of similarity metrics between two samples. The basic form of kernels for two sample vectors with *M *dimensions **x**_*i *_= (*x*_*i*1_,..., *x*_*iM*_) and **x**_*j *_= (*x*_*j*1_,..., *x*_*jM*_) is represented by an inner product function such as *K*(**x**_*i*_, **x**_*j*_) = *ϕ*(**x**_*i*_)·*ϕ*(**x**_*j*_). *ϕ*(·) means an arbitrary mapping of vectors to another space with generally different dimensions called 'reproducing kernel Hilbert space (RKHS)' [7], which has many properties common to those of the Euclidean space. For technical convenience, rather than defining the mapping functions *ϕ*(·) for **x**_*i *_and **x**_*j*_, the inner product forms, i.e., *K*(**x**_*i*_, **x**_*j*_) = *ϕ*(**x**_*i*_)·*ϕ*(**x**_*j*_) of the mappings of **x**_*i *_and **x**_*j *_are preferably used in practical calculation [7]. The function *K*(**x**_*i*_, **x**_*j*_) is called a kernel.

Three standard kernels popularly used in microarray studies are the linear kernel: *K*(**x**_*i*_, **x**_*j*_) = **x**_*i*_·**x**_*j*_, the polynomial kernel: *K*(**x**_*i*_, **x**_*j*_) = (**x**_*i*_·**x**_*j *_+ 1)^*D*^, and the RBF kernel: K(xi,xj)=exp⁡(−∑k=1M(xik−xjk)2/σ2)
 MathType@MTEF@5@5@+=feaafiart1ev1aaatCvAUfKttLearuWrP9MDH5MBPbIqV92AaeXatLxBI9gBaebbnrfifHhDYfgasaacH8akY=wiFfYdH8Gipec8Eeeu0xXdbba9frFj0=OqFfea0dXdd9vqai=hGuQ8kuc9pgc9s8qqaq=dirpe0xb9q8qiLsFr0=vr0=vr0dc8meaabaqaciaacaGaaeqabaqabeGadaaakeaacqWGlbWscqGGOaakieqacqWF4baEdaWgaaWcbaGaemyAaKgabeaakiabcYcaSiab=Hha4naaBaaaleaacqWGQbGAaeqaaOGaeiykaKIaeyypa0JagiyzauMaeiiEaGNaeiiCaaNaeiikaGIaeyOeI0YaaabmaeaacqGGOaakcqWG4baEdaWgaaWcbaGaemyAaKMaem4AaSgabeaakiabgkHiTiabdIha4naaBaaaleaacqWGQbGAcqWGRbWAaeqaaOGaeiykaKYaaWbaaSqabeaacqaIYaGmaaGccqGGVaWliiGacqGFdpWCdaahaaWcbeqaaiabikdaYaaaaeaacqWGRbWAcqGH9aqpcqaIXaqmaeaacqWGnbqta0GaeyyeIuoaaaa@544D@. All these kernels belong to the vectorial data kernel family that takes vectorial data for *N *samples as input, and we can fill all the (*i*, *j*) elements in the *N *× *N *kernel matrix with the specified kernel function.

Any kernel matrix generated from such kernel functions possesses a necessary property for SVM learning called *positive semidefiniteness *(see Appendix for details). If a kernel matrix is positive semidefinite, the mapped vectors *ϕ*(**x**) exist in the RKHS where the triangle inequalities among the mapped vectors are conserved. Our aim is to develop kernels that are robust to heterogeneous and noisy gene expression data. To this end, we first devise a new distance metric called *k*NND (detailed later) that can fulfill our requirements. However, unfortunately, the triangle inequalities are not conserved in the metric. To construct valid kernels from such distances, we introduce the following ME kernel algorithm.

### ME kernel with kNND denoising

#### ME kernel algorithm

The ME kernel was recently devised by Tsuda and Noble [12] to represent yeast metabolic and protein-protein interaction network (graph) structures. Unlike the standard vectorial kernels, the ME kernel does not have any pre-defined functions. Instead, given distance constraints, *D*_*ij*_, between samples, we obtain the ME kernel in matrix form, **K**, by basically solving the following optimization problem:

max⁡KH(K)=−tr(Klog⁡K)
 MathType@MTEF@5@5@+=feaafiart1ev1aaatCvAUfKttLearuWrP9MDH5MBPbIqV92AaeXatLxBI9gBaebbnrfifHhDYfgasaacH8akY=wiFfYdH8Gipec8Eeeu0xXdbba9frFj0=OqFfea0dXdd9vqai=hGuQ8kuc9pgc9s8qqaq=dirpe0xb9q8qiLsFr0=vr0=vr0dc8meaabaqaciaacaGaaeqabaqabeGadaaakeaadaWfqaqaaiGbc2gaTjabcggaHjabcIha4bWcbaacbeGae83saSeabeaakiabdIeaijabcIcaOiab=TealjabcMcaPiabg2da9iabgkHiTiabbsha0jabbkhaYjabcIcaOiab=TealjGbcYgaSjabc+gaVjabcEgaNjab=TealjabcMcaPaaa@42F3@

subject to:

tr(**K**) = 1, ||*ϕ*(**x**_*i*_) - *ϕ*(**x**_*j*_)||^2 ^≤ *D*_*ij*_

This optimization cannot be solved analytically; hence, we have implemented an efficient numerical algorithm for optimization (for technical details, see [21]). The function *H*(**K**) = - tr(**K**log**K**) is called von Neumann entropy of the kernel matrix **K **[12]. The first constraint tr(**K**) = 1 is necessary to avoid unlimited divergence of matrix **K**. Constraints ||*ϕ*(**x**_*i*_) - *ϕ*(**x**_*j*_)||^2 ^≤ *D *_*ij *_are given as prior knowledge. For example, we can give the constraint such that a particular pair of the mapped samples in the RKHS must not be distant. Regardless of the constraint values, von Neumann entropy of a kernel is always maximized so that the kernel matrix holds positive semidefiniteness [12]. Thus, one can use different gene sets in calculating *D*_*ij *_depending on *i *and *j *samples. The RBF kernel, in contrast, violates positive semidefiniteness if different gene sets are used to construct a kernel matrix using the above function, namely, by negating and exponentiating distance matrices *D*_*ij *_[21]. Intuitively, as shown in Figure 1, the ME kernel is built by enlarging the geometric distances among samples when the kernel entropy is maximized. Only related samples can stay near each other due to the constraints given as *D*_*ij*_. Matrices **K **that resulted after fully maximizing *H*(**K**) can be used for further kernel analysis methods such as SVM classification and kernel principal component analysis [24].

### kNND denoising method for ME kernel

The main issue addressed herein is how to handle missing or noisy values that exist in a large portion of a gene expression profile consisting of heterogeneous data. To effectively eliminate such spurious values without removing the entire gene, we devised the following simple method. Assume that we have a gene expression table (i.e., *M *genes × *N *samples matrix) where a sample contains *ν *(×100)% of noisy genes on average. In such a case, only 1 - *ν *of genes in that sample contain no noise. Therefore, for any pair of samples, the ratio of common genes not containing noise is expected to be (1 - *ν*)^2^. Based on this observation, we compute the distance between two samples **x**_*i *_and **x**_*j *_as follows: First, we compute the one-dimensional Euclidean distances *d*_*h*_= (*x*_*ih *_- *x*_*jh*_)^2 ^for *h *= 1, ... , *M *genes. Then, we select *k *= (1 - *ν*)^2 ^× *M *of one-dimensional Euclidean distances *d*_*h *_from the nearest (smallest) ones. Finally, we take the sum of the selected *d*_*h*_s as the distance between **x**_*i *_and **x**_*j*_. We refer to this method as *k-nearest neighbor gene distance *denoising (*k*NND denoising) method hereafter. For instance, if a sample with *M *= 100 feature genes contains *ν *= 15% of noisy values, *k *= (1 - 0.15)^2 ^× 100 ≃ 72 of the nearest distance genes out of the 100 feature genes are only considered in calculating *k*NNDs between samples.

Multiplying the above *k*NNDs by constant *G *for *N *samples, an *N × N *distance constraint matrix (*D*_*ij*_) is generated. Note that since the samples use different gene sets in *k*NND metric, positive semidefiniteness will not hold when directly imported to the RBF kernel function. Instead, however, when our ME kernel algorithm is applied and those *k*NNDs are used *as constraint*, the resulting kernel matrix holds positive semidefiniteness as well as reflects similarities between samples. Subsequently, we will train SVMs for the optimized 'ME' kernel with sample labels.

### Other distance-based kernels

For comparison, we tested two approaches to obtain a kernel matrix from distance matrix *D*_*ij*_. Both approaches are originally devised for conversion of a non-positive-semidefinite similarity matrix **S **into a kernel matrix. The first approach is to take **S**^*T*^**S **as a new kernel matrix. The kernel is sometimes called *empirical kernel mapping (EKM) *[16]. The second approach is to subtract the smallest negative eigenvalue of the similarity matrix **S **from its diagonal. We call it the *Saigo kernel *[17]. We obtain a similarity matrix from distance matrix *D*_*ij*_ via *S*_*ij *_= exp(*-D*_*ij*_/*σ *^2^).

### Singular value decomposition of vectorial kernels

As an alternative and conventional approach to noise reduction, singular value decomposition (SVD) is often used in many analytical studies including microarray analysis [25,26]. We use this method to denoise microarrays for comparison. Intuitively, SVD reduces dimensions of data to only informative ones, thus denoising values with regard to non-informative dimensions. More formally, genes with *N *expression values that are centered by means are reduced to the major *q *principal components by solving the eigenvectors of *M *genes × *N *samples matrix **A**_*M *× *N*_:

AM×N=UM×N⋅WN×N⋅VN×NT,
 MathType@MTEF@5@5@+=feaafiart1ev1aaatCvAUfKttLearuWrP9MDH5MBPbIqV92AaeXatLxBI9gBaebbnrfifHhDYfgasaacH8akY=wiFfYdH8Gipec8Eeeu0xXdbba9frFj0=OqFfea0dXdd9vqai=hGuQ8kuc9pgc9s8qqaq=dirpe0xb9q8qiLsFr0=vr0=vr0dc8meaabaqaciaacaGaaeqabaqabeGadaaakeaaieqacqWFbbqqdaWgaaWcbaGaemyta0Kaey41aqRaemOta4eabeaakiabg2da9iab=vfavnaaBaaaleaacqWGnbqtcqGHxdaTcqWGobGtaeqaaOGaeyyXICTae83vaC1aaSbaaSqaaiabd6eaojabgEna0kabd6eaobqabaGccqGHflY1cqWFwbGvdaqhaaWcbaGaemOta4Kaey41aqRaemOta4eabaGaeSy==7gaaOGaeiilaWcaaa@4D1E@

where **U**_*M *× *N *_is *M *× *N *column-orthogonal matrix (columns are called left singular vectors) and VN×NT
 MathType@MTEF@5@5@+=feaafiart1ev1aaatCvAUfKttLearuWrP9MDH5MBPbIqV92AaeXatLxBI9gBaebbnrfifHhDYfgasaacH8akY=wiFfYdH8Gipec8Eeeu0xXdbba9frFj0=OqFfea0dXdd9vqai=hGuQ8kuc9pgc9s8qqaq=dirpe0xb9q8qiLsFr0=vr0=vr0dc8meaabaqaciaacaGaaeqabaqabeGadaaakeaaieqacqWFwbGvdaqhaaWcbaGaemOta4Kaey41aqRaemOta4eabaGaeSy==7gaaaaa@3570@ is *N *× *N *orthogonal matrix (rows are called right singular vectors). The matrix **W**_*N *× *N *_is *N *× *N *diagonal matrix and 1/(*N *- 1)**W**^2 ^equals eigenvalues of the uncentered covariance matrix of **A**. We choose the largest *q *eigenvalues and replace other diagonal elements of **W **with 0, creating the **W**_*q *_matrix. Finally, we obtain the denoised matrix, **A**_*q*_, with **A**_*q *_= **U**·**W**_*q*_·**V**^*T*^. The vectorial data kernels are subsequently computed from the denoised matrix, **A**_*q*_.

For actual analysis, software called SVDMAN developed by Wall *et al*. [27] is used.

### Support vector machines

The SVMs [8] are well used for the classification of samples on the basis of their input (gene expression) values [2,28]. The basic form of SVMs is a binary classifier having a hyper-plane that distinguishes two distributions of *M*-dimensional vectors or samples from different classes. The hyper-plane **w**·*ϕ*(**x**_*i*_) + *b *is obtained by solving the following optimization problem:

min⁡w,b,ξi12‖w‖2+C∑i=1Nξi
 MathType@MTEF@5@5@+=feaafiart1ev1aaatCvAUfKttLearuWrP9MDH5MBPbIqV92AaeXatLxBI9gBaebbnrfifHhDYfgasaacH8akY=wiFfYdH8Gipec8Eeeu0xXdbba9frFj0=OqFfea0dXdd9vqai=hGuQ8kuc9pgc9s8qqaq=dirpe0xb9q8qiLsFr0=vr0=vr0dc8meaabaqaciaacaGaaeqabaqabeGadaaakeaadaWfqaqaaiGbc2gaTjabcMgaPjabc6gaUbWcbaacbeGae83DaCNaeiilaWIaemOyaiMaeiilaWccciGae4NVdG3aaSbaaWqaaiabdMgaPbqabaaaleqaaOWaaSaaaeaacqaIXaqmaeaacqaIYaGmaaWaauWaaeaacqWF3bWDaiaawMa7caGLkWoadaahaaWcbeqaaiabikdaYaaakiabgUcaRiabdoeadnaaqahabaGae4NVdG3aaSbaaSqaaiabdMgaPbqabaaabaGaemyAaKMaeyypa0JaeGymaedabaGaemOta4eaniabggHiLdaaaa@4C8E@

subject to

*y*_*i*_(**w**·*ϕ *(**x**_*i*_) + *b*) ≥ 1 - *ξ*_*i*_, *ξ*_*i*_≥ 0

for all *N *samples where *y*_*i *_and *C *are the class label of *i*-th sample and a constant parameter, respectively. Optimization of this problem yields the hyper-plane that maximizes the margin between the two classes. This is called the SVM learning algorithm. The above optimization problem can be transformed into an equivalent form of the other equations in which only the kernel function *K*(**x**_*i*_, **x**_*j*_) = *ϕ*(**x**_*i*_)·*ϕ*(**x**_*j*_) appears and mapped vectors *ϕ*(**x**_*j*_) are not explicitly described [7]. Hence, the SVM learning algorithm needs only kernel matrix **K **and mapped vectors *ϕ*(**x**_*j*_) are not necessary.

### Leave-one-out cross validation

The classification accuracies for the evaluation of the ME kernel against other methods are estimated by a standard leave-one-out cross-validation (LOOCV) procedure where each sample is alternatively excluded from the *N *data and the SVM trained with the remaining *N - *1 samples predicts the excluded one. All accuracies reported in this paper are calculated with the following formula:

Accuracy=TP+TNTP+FP+TN+FN
 MathType@MTEF@5@5@+=feaafiart1ev1aaatCvAUfKttLearuWrP9MDH5MBPbIqV92AaeXatLxBI9gBaebbnrfifHhDYfgasaacH8akY=wiFfYdH8Gipec8Eeeu0xXdbba9frFj0=OqFfea0dXdd9vqai=hGuQ8kuc9pgc9s8qqaq=dirpe0xb9q8qiLsFr0=vr0=vr0dc8meaabaqaciaacaGaaeqabaqabeGadaaakeaacqWGbbqqcqWGJbWycqWGJbWycqWG1bqDcqWGYbGCcqWGHbqycqWGJbWycqWG5bqEcqGH9aqpdaWcaaqaaiabdsfaujabdcfaqjabgUcaRiabdsfaujabd6eaobqaaiabdsfaujabdcfaqjabgUcaRiabdAeagjabdcfaqjabgUcaRiabdsfaujabd6eaojabgUcaRiabdAeagjabd6eaobaaaaa@49C0@

where *TP*, *FP*, *TN *and *FN *are true positive, false positive, true negative, and false negative frequencies, respectively, in the classification.

### Parameter selection

Since classification accuracies are dependent on parameters in the kernel-SVM method, we tested various parameter values to obtain the best performance possible. For all the six (linear, polynomial, RBF, EKM, Saigo, and ME) kernels tested here, seven SVM parameters, *C *= {10^-3^, 10^-2^, 10^-1^, 1,10, 10^2^, 10^3^}, are tested. For the polynomial kernel, *D *= {1, 2, 3, 4, 5, 6, 7, 8, 9, 10} are tested. For the RBF, EKM, and Saigo kernels, *σ *= {10^-10^, 10^-9^, 10^-8^,10^-7^, 10^-6^, 10^-5^,10^-4^, 10^-3^, 10^-2^,10^-1^, 1} are tested. In the ME kernel, we used only one parameter *G *that magnifies the distance constraints *D*_*ij *_to adjust the trade-off between over-learning and generalization of classification models (for details, see [21]). The parameter *G *has to be chosen carefully. When *G *→ 0, typically **K **→ **11**^⊤^/*N*. When *D*_*ij *_> 2/*N *for ∀*i*, ∀*j*, **K **→ **I**/*N*. The two are somewhat extreme cases. However, if the value of *G *is positive but too small, SVM cannot find the hyper-plane separating the positive class from the negative one clearly. If the value of *G *is too large, it leads to the so-called diagonal dominant problem [16]. We tested the parameter in the range of *G *= {2^-5^, 2^-4^, 2^-3^, 2^-2^, 2^-1^, 1, 2, 2^2^, 2^3^, 2^4^, 2^5^}. Note that the number of parameter combinations in the ME kernel is equal to those in the RBF, EKM and Saigo kernels in this study.

## Authors' contributions

WF conceived of the study, carried out the analyses and drafted the manuscript. TK participated in the design of the study, wrote the program and helped to draft the manuscript. Both authors have read and approved the final manuscript.

## Appendix: positive semidefiniteness of kernels

Formally, the positive semidefiniteness of a kernel matrix **K**, which guarantees the existence of mapping functions *ϕ *(·) for sample vectors, **x**_*i *_and **x**_*j*_, is defined as follows: A symmetric matrix **K **is said to be *positive semidefinite *if **K **holds

∑i,jcicjKij≥0
 MathType@MTEF@5@5@+=feaafiart1ev1aaatCvAUfKttLearuWrP9MDH5MBPbIqV92AaeXatLxBI9gBaebbnrfifHhDYfgasaacH8akY=wiFfYdH8Gipec8Eeeu0xXdbba9frFj0=OqFfea0dXdd9vqai=hGuQ8kuc9pgc9s8qqaq=dirpe0xb9q8qiLsFr0=vr0=vr0dc8meaabaqaciaacaGaaeqabaqabeGadaaakeaadaaeqbqaaiabdogaJnaaBaaaleaacqWGPbqAaeqaaOGaem4yam2aaSbaaSqaaiabdQgaQbqabaGccqWGlbWsdaWgaaWcbaGaemyAaKMaemOAaOgabeaaaeaacqWGPbqAcqGGSaalcqWGQbGAaeqaniabggHiLdGccqGHLjYScqaIWaamaaa@3EDF@

for any real numbers, *c*_*i *_and *c*_*j*_.

Practically, a symmetric matrix **K **is positive semidefinite if and only if a mapped feature vector can be assigned to each sample such that each element of the matrix satisfies *K*_*ij *_= *ϕ*(**x**_*i*_)·*ϕ*(**x**_*j*_), where *ϕ*(**x***i*) and *ϕ*(**x**_*j*_) are the mapped feature vectors of *i*-th sample and *j*-th sample, respectively [29]. For example, a symmetric matrix

K=[1641245912918]
 MathType@MTEF@5@5@+=feaafiart1ev1aaatCvAUfKttLearuWrP9MDH5MBPbIqV92AaeXatLxBI9gBaebbnrfifHhDYfgasaacH8akY=wiFfYdH8Gipec8Eeeu0xXdbba9frFj0=OqFfea0dXdd9vqai=hGuQ8kuc9pgc9s8qqaq=dirpe0xb9q8qiLsFr0=vr0=vr0dc8meaabaqaciaacaGaaeqabaqabeGadaaakeaaieqacqWFlbWscqGH9aqpdaWadaqaauaabeqadmaaaeaacqaIXaqmcqaI2aGnaeaacqaI0aanaeaacqaIXaqmcqaIYaGmaeaacqaI0aanaeaacqaI1aqnaeaacqaI5aqoaeaacqaIXaqmcqaIYaGmaeaacqaI5aqoaeaacqaIXaqmcqaI4aaoaaaacaGLBbGaayzxaaaaaa@3D60@

is positive semidefinite since we can assign mapped feature vectors as:

*ϕ*(**x**_1_) = (0, 4)^⊤^, *ϕ*(**x**_2_) = (2, 1)^⊤^, *ϕ*(**x**_3_) = (3, 3)^T^,

which hold *K*_*ij *_= *ϕ*(**x**_*i*_)·*ϕ*(**x**_*j*_) indeed. A symmetric matrix,

K=[164124591295]
 MathType@MTEF@5@5@+=feaafiart1ev1aaatCvAUfKttLearuWrP9MDH5MBPbIqV92AaeXatLxBI9gBaebbnrfifHhDYfgasaacH8akY=wiFfYdH8Gipec8Eeeu0xXdbba9frFj0=OqFfea0dXdd9vqai=hGuQ8kuc9pgc9s8qqaq=dirpe0xb9q8qiLsFr0=vr0=vr0dc8meaabaqaciaacaGaaeqabaqabeGadaaakeaaieqacqWFlbWscqGH9aqpdaWadaqaauaabeqadmaaaeaacqaIXaqmcqaI2aGnaeaacqaI0aanaeaacqaIXaqmcqaIYaGmaeaacqaI0aanaeaacqaI1aqnaeaacqaI5aqoaeaacqaIXaqmcqaIYaGmaeaacqaI5aqoaeaacqaI1aqnaaaacaGLBbGaayzxaaaaaa@3C6A@

is not positive semidefinite since there exist no mapped feature vectors satisfying *K*_*ij *_= *ϕ*(**x**_*i*_)·*ϕ*(**x**_*j*_).

## References

[B1] Golub TR, Slonim DK, Tamayo P, Huard C, Gaasenbeek M, Mesirov JP, Coller H, Loh ML, Downing JR, Caligiuri MA, Bloomfield CD, Lander ES (1999). Molecular Classification of Cancer: Class Discovery and Class Prediction by Gene Expression Monitoring. Science.

[B2] Ramaswamy S, Tamayo P, Rifkin R, Mukherjee S, Yeang C, Angelo M, Ladd C, Reich M, Latulippe E, Mesirov JP, Poggio T, Gerald W, Loda M, Lander ES, Golub TR (2001). Multiclass cancer diagnosis using tumor gene expression signatures. Proc Natl Acad Sci USA.

[B3] Staunton JE, Slonim DK, Coller HA, Tamayo P, Angelo MJ, Park J, Scherf U, Lee JK, Reinhold WO, Weinstein JN, Mesirov JP, Lander ES, Golub TR (2001). Chemosensitivity prediction by transcriptional profiling. Proc Natl Acad Sci USA.

[B4] Okutsu J, Tsunoda T, Kaneta Y, Katagiri T, Kitahara O, Zembutsu H, Yanagawa R, Miyawaki S, Kuriyama K, Kubota N, Kimura Y, Kubo K, Yagasaki F, Higa T, Taguchi H, Tobita T, Akiyama H, Takeshita A, Wang YH, Motoji T, Ohno R, Nakamura Y (2002). Prediction of chemosensitivity for patients with acute myeloid leukemia, according to expression levels of 28 genes selected by genome-wide complementary DNA microarray analysis. Mol Cancer Ther.

[B5] van't Veer LJ, Dai H, van de Vijver MJ, He YD, Hart AAM, Mao M, Peterse HL, van der Kooy K, Marton MJ, Witteveen AT, Schreiber GJ, Kerkhoven RM, Roberts C, Linsley PS, Bernards R, Friend SH (2002). Gene expression profiling predicts clinical outcome of breast cancer. Nature.

[B6] Liu H, Li J, Wong L (2005). Use of extreme patient samples for outcome prediction from gene expression data. Bioinformatics.

[B7] Cristianini N, Shawe-Taylor J (2000). An Introduction to Support Vector Machines and Other Kernel-Based Learning Methods.

[B8] Vapnik V (1998). Statistical Learning Theory.

[B9] Lodhi H, Saunders C, Shawe-Taylor J, Cristianini N, Watkins C (2002). Text classification using string kernels. The Journal of Machine Learning Research.

[B10] Tsuda K, Kin T, Asai K (2002). Marginalized kernels for biological sequences. Bioinformatics.

[B11] Kondor R, Lafferty J, Sammut C, Hoffmann AG (2002). Diffusion kernels on graphs and other discrete structures. Proc 19th Intl Conf on Machine Learning (ICML) [ICML 2002].

[B12] Tsuda K, Noble WS (2004). Learning kernels from biological networks by maximizing entropy. Bioinformatics.

[B13] Rhodes DR, Yu J, Shanker K, Deshpande N, Varambally R, Ghosh D, Barrette T, Pandey A, Chinnaiyan AM (2004). Large-scale meta-analysis of cancer microarray data identifies common transcriptional profiles of neoplastic transformation and progression. Proc Natl Acad Sci USA.

[B14] Warnat P, Eils R, Brors B (2005). Cross-platform analysis of cancer microarray data improves gene expression based classification of phenotypes. BMC Bioinformatics.

[B15] Nilsson B, Andersson A, Johansson M, Fioretos T (2006). Cross-platform classification in microarray-based leukemia diagnostics. Haematologica.

[B16] Scholköpf B, Weston J, Eskin E, Leslie C, Noble WS (2002). A Kernel Approach for Learning From Almost Orthogonal Patterns. Proceedings of ECML 2002i, 13th European Conference on Machine Learning.

[B17] Saigo H, Vert JP, Ueda N, Akutsu T (2004). Protein homology detection using string alignment kernels. Bioinformatics.

[B18] Supplemental datasets in this paper. http://cellmontage.cbrc.jp/~wataru/ME/.

[B19] Rosner B (2000). Fundamentals of Biostatistics.

[B20] Barrett T, Suzek TO, Troup DB, Wilhite SE, Ngau WC, Ledoux P, Rudnev D, Lash AE, Fujibuchi W, Edgar R (2005). NCBI GEO: mining millions of expression profiles-database and tools. Nucleic Acids Res.

[B21] Kato T, Fujibuchi W, Asai K (2006). Kernel Analysis for Noisy Microarray Data. AIST Technical Report.

[B22] O'Donnell RK, Kupferman M, Wei SJ, Singhal S, Weber R, Jr BO, Cheng Y, Putt M, Feldman M, Ziober B, Muschel RJ (2005). Gene expression signature predicts lymphatic metastasis in squamous cell carcinoma of the oral cavity. Oncogene.

[B23] Torunera GA, Ulgera C, Alkana M, Galanted AT, Rinaggioe J, Wilkf R, Tiang B, Soteropoulosa P, Hameedh MR, Schwalba MN, Dermody JJ (2004). Association between gene expression profile and tumor invasion in oral squamous cell carcinoma. Cancer Genet Cytogenet.

[B24] Liu Z, Chen D, Bensmail H (2005). Gene expression data classification with kernel principal component analysis. J Biomed Biotechnol.

[B25] Alter O, Brown PO, Botstein D (2000). Singular value decomposition for genome-wide expression data processing and modeling. Proc Natl Acad Sci USA.

[B26] Liu L, Hawkins DM, Ghosh S, Young SS (2003). Robust singular value decomposition analysis of microarray data. Proc Natl Acad Sci USA.

[B27] Wall ME, Dyck PA, Brettin TS (2001). SVDMAN-singular value decomposition analysis of microarray data. Bioinformatics.

[B28] Tothill RW, Kowalczyk A, Rischin D, Bousioutas A, Haviv I, van Laar RK, Waring PM, Zalcberg J, Ward R, B AV, Sutherland RL, Henshall SM, Fong K, Pollack JR, Bowtell DDL, Holloway AJ (2005). An Expression-Based Site of Origin Diagnostic Method Designed for Clinical Application to Cancer of Unknown Origin. Cancer Res.

[B29] Schölkopf B, Smola AJ (2001). Learning with Kernels.

[B30] Boer JM, Huber WK, Sültmann H, Wilmer F, von Heydebreck A, Haas S, Korn B, Gunawan B, Vente A, Füzesi L, Vingron M, Poustka A (2001). Identification and Classification of Differentially Expressed Genes in Renal Cell Carcinoma by Expression Profiling on a Global Human 31,500-Element cDNA Array. Genome Res.

[B31] Su AI, Cookedagger MP, Chingdagger KA, Hakakdagger Y, Walkerdagger JR, Wiltshiredagger T, Orthdagger AP, VegaDagger RG, SapinosoDagger LM, Moqrich A, Patapoutian A, HamptonDagger GM, Schultz PG, Hogenesch JB (2002). Large-scale analysis of the human and mouse transcriptomes. Proc Natl Acad Sci USA.

[B32] Yanai I, Benjamin H, Shmoish M, Chalifa-Caspi V, Shklar M, Ophir R, Bar-Even A, Horn-Saban S, Safran M, Domany E, Lancet D, Shmueli O (2005). Genome-wide midrange transcription profiles reveal expression level relationships in human tissue specification. Bioinformatics.

[B33] Flechnera SM, Kurianb SM, Headc SR, Sharpb SM, Whisenantc TC, Zhangd J, Chismarc JD, Horvathe S, Mondalac T, Gilmartinc T, Cooka DJ, Kayd SA, Walkerd JR, Salomon DR (2004). Kidney Transplant Rejection and Tissue Injury by Gene Profiling of Biopsies and Peripheral Blood Lymphocyte. Am J Transplant.

[B34] Su AI, Wiltshire T, Batalov S, Lapp H, Ching KA, Block D, Zhang J, Soden R, Hayakawa M, Kreiman G, Cooke MP, Walker JR, Hogenesch JB (2004). A gene atlas of the mouse and human protein-encoding transcriptomes. Proc Natl Acad Sci USA.

[B35] Lenburg ME, Liou LS, Gerry NP, Frampton GM, Cohen HT, Christman MF (2003). Previously unidentified changes in renal cell carcinoma gene expression identified by parametric analysis of microarray data. BMC Cancer.

